# A new MOF-based modified adsorbent for the efficient removal of Hg(ii) ions from aqueous media: isotherms and kinetics

**DOI:** 10.1039/d4ra00770k

**Published:** 2024-05-22

**Authors:** Hamid Reza Sobhi, Mojtaba Yeganeh, Mahnaz Ghambarian, Sevda Fallah, Ali Esrafili

**Affiliations:** a Department of Chemistry, Payame Noor University Tehran Iran; b Research Center for Environmental Health Technology, Iran University of Medical Sciences Tehran Iran a.esrafili@iums.ac.ir a_esrafily@yahoo.com; c Department of Environmental Health Engineering, School of Public Health, Iran University of Medical Sciences Tehran Iran; d Iranian Research and Development Center for Chemical Industries, ACECR Tehran Iran; e Department of Environmental Health Engineering, Student Research Committee, School of Public Health and Safety, Shahid Beheshty University of Medical Science Tehran Iran

## Abstract

Herein, a new MOF-based modified adsorbent for the efficient removal of Hg(ii) ions from water media was successfully prepared. Initially, a MOF nanocomposite was synthesized and applied as an efficient adsorbent for the removal of the target heavy metal ion. Following the synthesis, the MOF-based modified adsorbent was identified and characterized by SEM, XRD and FT-IR analytical instruments. The impact of several key variables such as pH of aqueous solution, adsorbent dosage, contact time, and initial concentration of the analyte of interest on the adsorption efficiency was also investigated in detail. Under the optimal conditions established (pH, 3; dose of adsorbent, 0.4 g L^−1^; contact time, 40 min and the analyte's concentration of 1 mg L^−1^) the removal efficiency of 96.3% for Hg(ii) was obtained. The results of the studies on the isotherm and kinetics of adsorption revealed that the adsorption process of Hg(ii) matched with the Langmuir isotherm (*R*^2^ > 0.990) and the pseudo 2nd-order kinetic models (*R*^2^ > 0.998). Additionally, reuse of the applied adsorbent for five consecutive tests exhibited a small percentage of drop (about 8%) in the removal efficiency of the target ion. Finally, the results indicated that the MOF-based modified compound could be potentially applied as a highly efficacious adsorbent for the discharge of Hg(ii) from aquatic media.

## Abstract 2

Herein, a new MOF-based modified adsorbent for the efficient removal of Hg(ii) ions from water media was successfully prepared. This compound has potential as a highly efficacious adsorbent for the discharge of Hg(ii) from aquatic media.

## Graphical abstract

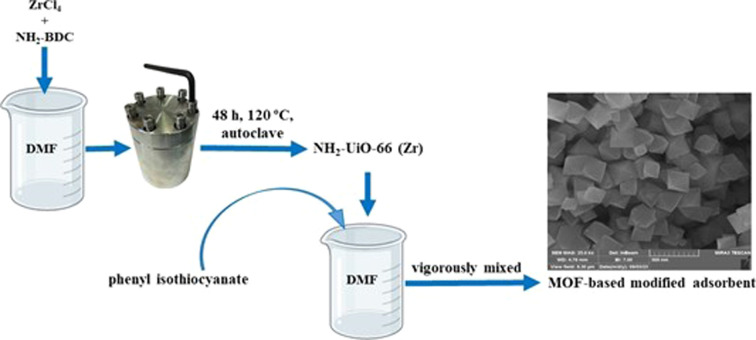

## 1. Introduction

Generally, environmental pollution is considered as one of the great challenges of our time. The significant growth of industrial activities along with the expansion of urban life has caused the release of a large amount of polluting and toxic substances into aquatic media. Industrial-based effluents normally contain high level of toxic and dangerous compounds including pharmaceuticals, hygienic products, dyes, pesticides and heavy metals, to name but a few.^1–3^

Heavy metals have been regarded as being one of the most severe environmental issues these days. Metal ions, especially heavy metal ones, are extremely dangerous and compared to organic compounds they have a long shelf life and easily end up in the food chain and ultimately the human body. It is worth noting that heavy metals are not easily degraded on account of their complicated bioaccumulation features.^4–6^ Therefore, the removal of these metals are detrimental to the protection of human being and the environment.

Up to now, a various number of extraction/separation methods have been implemented for the discharge of these ions, namely ion exchange, precipitation, adsorption techniques, filtration, electrochemical techniques, *etc.*^7–9^ Recently, the implication of proper and suitable adsorbents for the extraction and removal of these pollutants has been enormously focused on. In brief, the advantages of adsorption process compared to other methods are as follows: low cost, wide range of applications, simple design, ease of operation, less production of secondary toxics and high level of recovery and efficiency.^10–12^

Till now, wide variety of adsorbents have been employed to reduce and ultimately remove pollutants from the environment including mesoporous compounds, activate carbon, zeolite, and metal–organic frameworks (MOFs).^13–15^ Amongst them, porous MOFs have received enormous attention and applied in this field because of their various applications. It should be highlighted that MOFs are preferred over other porous compounds due to high porosity with tunability, functionalization and large accessibility of metal sites, *etc.*^16,17^

Co-ordinated polymers are complicated structures that are made from primary building units including organic ligands and metal ions. These primary constituent units are connected by coordination bonds and other weak/reversible chemical bonds. These three-dimensional coordination polymers with stability and permanent porosity are placed in the category with the specific name of MOFs. The possibility of designing a high porosity structure, inherent crystallinity and cavities with different shape and size is among the important distinguishing features of MOFs.^18–20^

Therefore, extensive reports on the adsorption and removal of various toxic compounds using the MOF-based structures. It is highlighted that various probable interactions namely, electrostatic, acid–base, π–π stacking and hydrogen bonding, *etc.* have to be considered (between the MOF sites and pollutant) to understand and elucidate the mechanism of adsorption.^21^

On the other hand, the most distinctive feature of MOFs compared to other porous compounds is the possibility of decorating their constituent units with different functional groups. The presence of metal centers and organic binders are highly appropriate to introduce a various number of functional groups. The active metal centers (serving as the Lewis acids) are capable of establishing strong and selective bonds with different molecules. The introduction of suitable functional groups to the MOF structure provides further accessible sites, on which, the guest molecules/ions can be adsorbed through various interactions mentioned earlier. This ultimately improves the performance and efficiency of the MOF structure with regard to adsorption process. Having said that, the construction of the MOFs with special functional groups results in the production of multivariate MOFs, which has been regarded as one of the most challenging issues in the synthetic process.^22–24^

As mentioned earlier, one of the major superiorities of the MOF-based structures is to design and locate desirable functional groups inside the cavities, which maximizes the interactions and leads to high diffusion and fast adsorption of the pollutants including heavy metals. For this reason and to meet the above objective, the applied MOF with several excellent features such as high surface area and properly oriented cavities, was initially synthesized and then modified with phenyl isothiocyanate (serving as the modifier agent). The resulting MOF-based modified compound was applied as a highly suitable adsorbent for the adsorption of a prominent ion with high grade of toxicity (Hg(ii)), already proven to be extremely harmful to human and environment even at low concentration levels. To best of our knowledge, no report on the preparation and implication of the applied adsorbent for the removal of Hg(ii) ions has been published yet.

## 2. Experimental

### 2.1. Chemicals and instruments

Zirconium chloride (ZrCl_4_), mercury nitrate (Hg(NO_3_)_2_), 2-aminoterephthalic acid (NH_2_-BDC), HCl, NaOH, absolute ethanol (EtOH), dimethyl formamide (DMF) and phenyl isothiocyanate were supplied by Merck Company (Darmstadt, Germany). Pure deionized water (DI-water) was applied to prepare the aqueous solutions and the solution pH was set with 0.1 M NaOH and/or 0.1 M HCl reagents. It is highlighted that all experiments were carried out at ambient condition. In addition, the following analytical instruments were employed to fully characterize the structure of the functionalized MOF-based adsorbent.

FT-IR spectrometer (Nicolet IR 100) was applied to identify the functional group of the adsorbent. X-ray diffractometer (XRD) with a copper lamp (Cu, Kα: *λ* = 1.54 Å; Philips Xpert) was used to characterize the crystalline structure. Scanning electron microscope (FE-SEM) (TESCAN MIRA) was used to determine the morphology of the adsorbent as well. ICP-MS instrument (PerkinElmer 9000) was implemented for the measurement of Hg(ii) ions.

### 2.2. Synthesis of the NH_2_-UiO-66 (Zr)

The mixture containing ZrCl_4_ (0.175 g) and NH_2_-BDC (0.136 g) were dispersed in DMF (38 mL) and heated up to 120 °C for 48 h in an autoclave and finally cooled down to ambient condition. As a result, MOF (NH_2_-UiO-66 (Zr)) was obtained and rinsed with DMF and air-dried.

### 2.3. Modification of NH_2_-UiO-66 (Zr)

For the modification of the synthesized MOF (NH_2_-UiO-66 (Zr)), 1.1 g of the above MOF was vigorously mixed with 2.6 g of phenyl isothiocyanate (modifier agent) in 25 mL DMF in a proper vessel for 15 min at ambient condition. Following that, the resulting precipitate (MOF-based modified adsorbent) was withdrawn and gently washed with DMF and ethanol and subsequently dried.

### 2.4. Adsorption procedure

In the current study, 100 mL of aqueous solution (placed in a 250 mL conical flask serving as a reactor) was agitated on a shaker-incubator at a fixed rate and used to conduct the experiments throughout. Furthermore, solution pH (1–9), dose of adsorbent (0.1–0.6 g L^−1^), Hg(ii) concentration (1–4 mg L^−1^) and contact time (0–90 min) were initially chosen as the potentially effective parameters. The value of the equilibrium adsorption capacity (*Q*_e_ (mg g^−1^)) was measured adopting the eqn (1):1
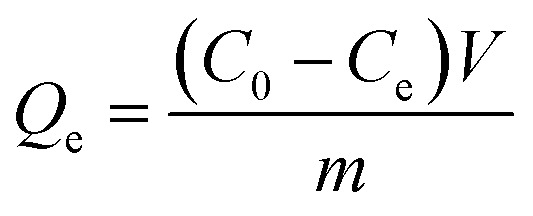
*C*_0_ and *C*_e_ denote as the initial and equilibrium concentration of Hg(ii) ions in the solution (mg L^−1^), *V* represents the volume of the solution (L) and *m* is the amount of the adsorbent used (g).

In addition, to evaluate the isotherm of adsorption all the effective variables are adjusted in such a way that they are at their optimal values. Finally, the kinetic study on Hg(ii) adsorption was assessed at room temperature and under the optimal conditions set.

## 3. Results and discussion

### 3.1. Adsorbent characteristics

**Fig. 1:**
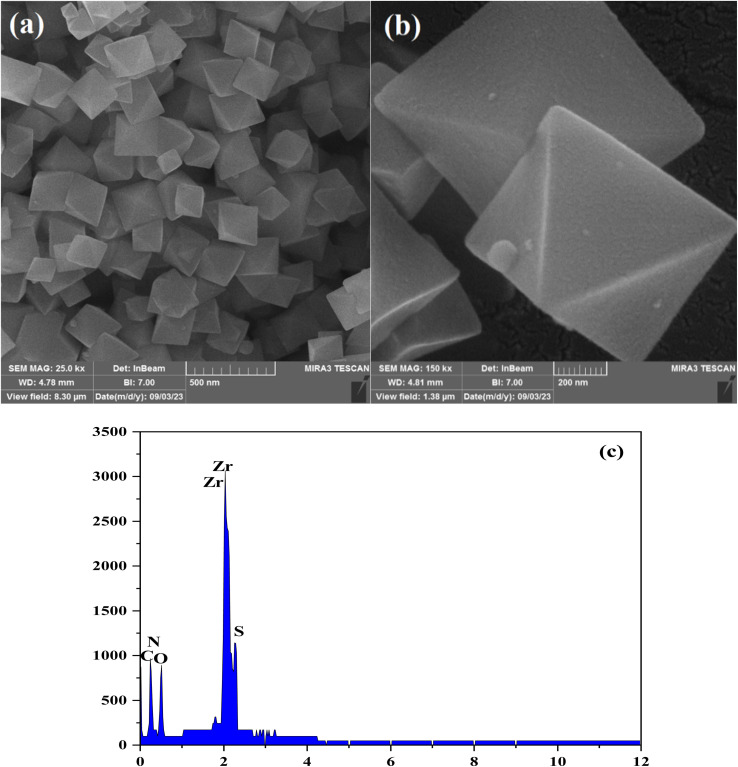
(a and b) SEM images and (c) and EDAX analysis.

The SEM instrument was employed to determine and identify the morphology of the MOF-based modified adsorbent. The SEM images obtained clarified that the adsorbent structure is of octahedral shape (see Fig. 1(a) and (b)). Moreover, the structural purity of MOF-based modified adsorbent was tested by the EDAX analysis (Fig. 1(c)). As depicted in the EDAX graph (Fig. 1(c)), MOF-based modified adsorbent is made up of C, N, O, S and Zr elements.

**Fig. 2:**
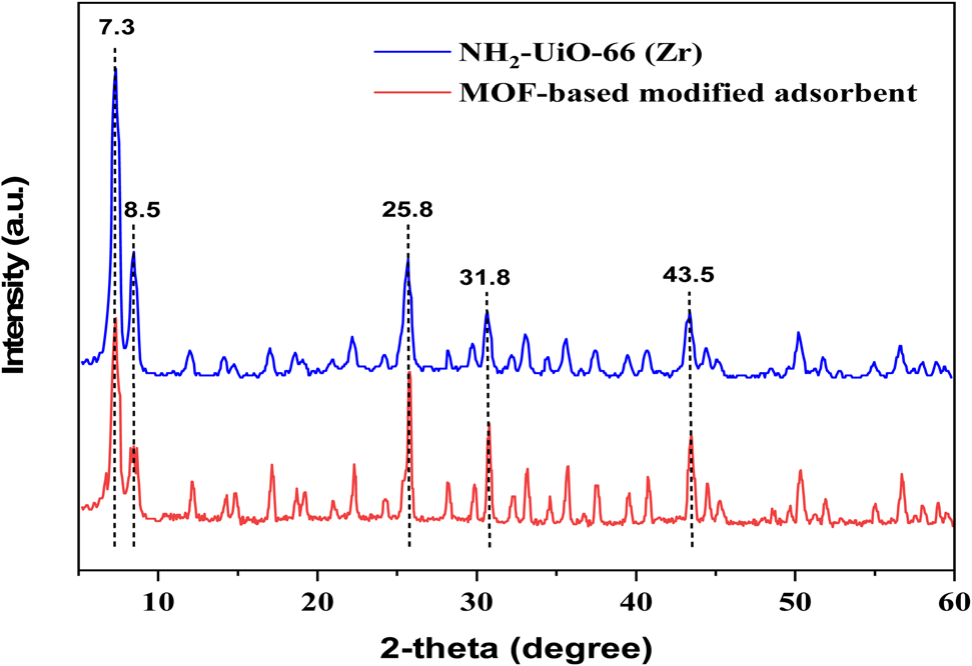
XRD spectra of the NH_2_-UiO-66 (Zr) and MOF-based modified adsorbent.

The XRD experiments were conducted to identify the chemical structure in terms of phase analysis as well as the degree of crystalline of the synthesized nanocomposite. The XRD patterns of the nanocomposite are presented in Fig. 2.

The identifying diffraction peaks pinpointed at 7.3°, 8.5°, 25.8°, 31.8° and 43.5° were in agreement with that of NH_2_-UiO-66 (Zr) previously reported elsewhere. The Zr-MOF pattern illustrates the intensive peaks at about 2*θ* of 7.3 and 8.5° ascribed to the (111) and the (200) tetragonally positioned planes of zirconia, respectively. The sharp peaks within the XRD patterns of the samples are indicative of desirable degree of crystallinity.^25,26^ The XRD results demonstrate that the diffraction pattern of the MOF-based modified adsorbent is well fitted to the corresponding reference (see Fig. 2).

As a rule of thumb, designing and introducing special functional groups onto MOFs significantly improves their efficiency for the adsorption of specific pollutants. Amongst the neutral factionalizing precursor chemicals used for the modification of MOFs, the ones donating the proper functional groups to the analytes of interest through various polar/apolar interactions are of high interest. In this particular study, the modification of the synthesized MOF (NH_2_-UiO-66 (Zr)) with phenyl isothiocyanate agent provides very ideal and unique conditions for host–guest interactions through the formation of –NH and –C=S donating groups. This facilitates the suitable coordination sites for Hg(ii) adsorption.

**Fig. 3:**
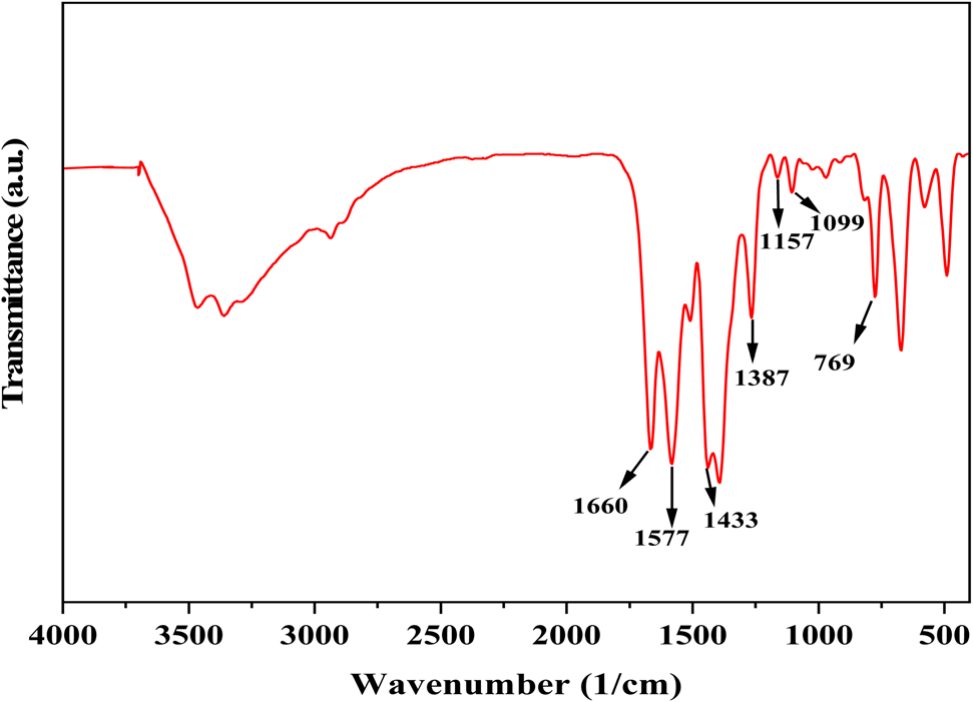
FT-IR spectrum of the MOF-based modified adsorbent.

In a further structurally-related test, the spectrum of FT-IR of the MOF-based modified adsorbent is depicted in Fig. 3. Briefly, the relatively broad band seen at the region of 3000–3050 cm^−1^ is likely to be related to the vibrating mode of the aromatic –C–H bonds. Whilst, the bands shown at 3350–3450 cm^−1^ might be referred to the vibration of the hydroxyl group –O–H and –N–H groups. The peak at the 1660 cm^−1^ is highly likely the prominent peak for the vibrational –C=S bond. The sharp peaks shown at 1387 and 1433 cm^−1^ could be related to the aromatic ring. The absorption band at 1577 cm^−1^ might be ascribed to –N–H group. The other peaks within the FT-IR spectrum shown at 1157, 1099, and 769 cm^−1^ could be originated from the vibrational –C–O, –C–N and bending mode of –C=S bonds. On the whole, the FT-IR analysis is indicative of the fact that the applied the MOF-based modified adsorbent was successfully synthesized.

**Fig. 4:**
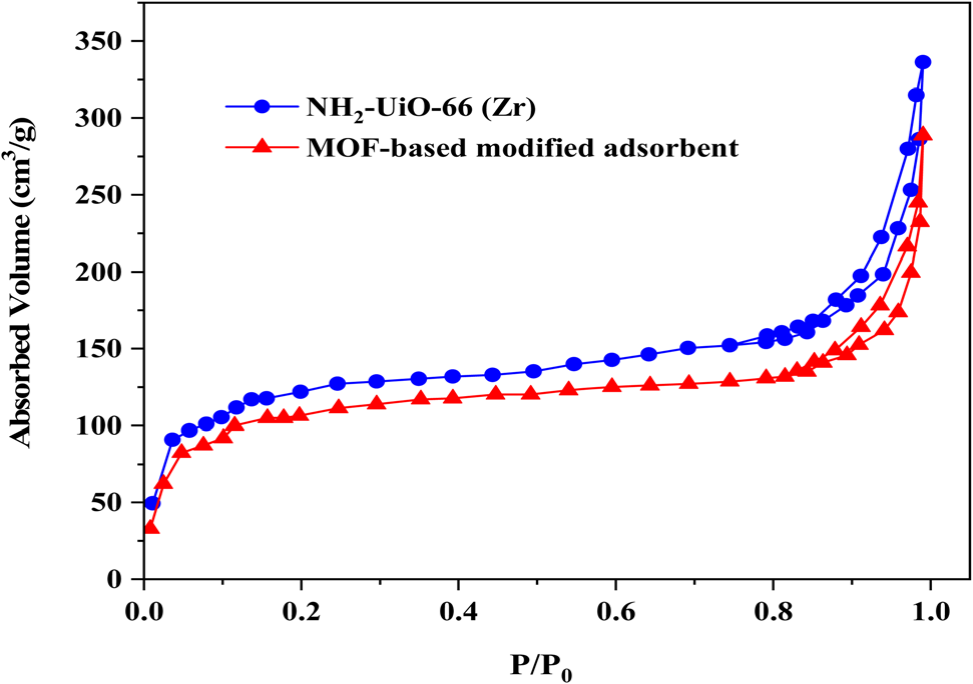
N_2_ adsorption–desorption isotherms for NH_2_-UiO-66 (Zr) and MOF-based modified adsorbent.

In a further related experiment, the specific surface area of UiO-66-NH_2_ and MOF-based modified adsorbent were measured by N_2_ adsorption–desorption isotherms (Fig. 4). Accordingly, the respective BET surface area of UiO-66-NH_2_ and MOF-based modified adsorbent were calculated to be 873.79 and 781.61 m^2^ g^−1^, respectively.

### 3.2. Effect of solution pH

**Fig. 5:**
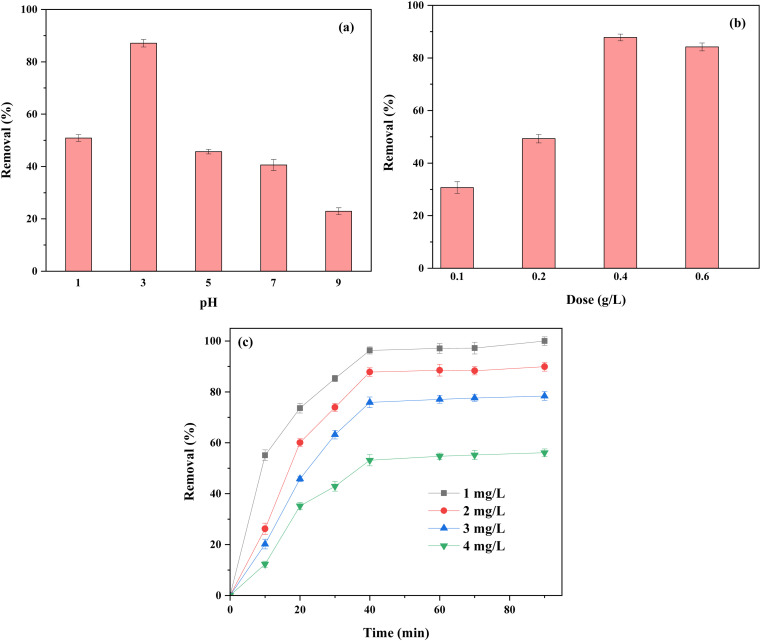
The effect of pH (a), adsorbent dosage (b), contact time and concentration (c) of Hg(ii) ions on the adsorption efficiency.

It is evident that solution pH is a key parameter in the adsorption of Hg(ii) ions from aquatic media. Furthermore, pH of the solution determines the presence of different forms of ions and the degree of ionization of the functional groups located on the modified adsorbent surface. To assess the impact of pH on Hg(ii) adsorption, the pH of the aqueous solution was altered within the 1–9 pH range and the respective results demonstrated that the highest level of adsorption for the studied heavy metal was observed at pH 3 (see Fig. 5(a)). At low pH values, the dominant species in the aqueous solution for the studied ion (Hg(ii)) is Hg^2+^, which can be efficiently adsorbed by the applied adsorbent. Contrarily, with an increase in the pH values Hg(OH)_2_ and possibly HgO become the prominent species in the solution, resulting in the drop in the efficiency of the adsorption.

### 3.3. Effect of adsorbent dosage

In a further related experiment, the adsorption efficiency of Hg(ii) on the applied adsorbent using different adsorbent dosages (0.1–0.6 g L^−1^) were evaluated. Fig. 5(b) demonstrates that by elevating the dose of adsorbent (from 0.1 to 0.4 g L^−1^), the adsorption efficiencies for the target metal ion improved and afterward levelled off. It seems that the combination of high surface area, proper cavities located on the applied adsorbent as well as the adsorbent's donating groups, are highly likely responsible for high level of increases in the adsorption efficiencies for the metal ion of interest.^27^

### 3.4. Effects of analyte's concentration and contact time

Fig. 5(c) demonstrates as to how the variation in the contact time spanning within 0–90 min can affect the adsorption efficiency. The adsorption process proceeded relatively fast (for 30 min) and then fell down gradually until the state of equilibrium was reached (approximately at 40 min). The swift adsorption efficiency shown in the graph shown is probably related to the high concentration of Hg(ii) ions and also the availability of further surface area on the adsorbent. As time elapsed (over 40 min), the adsorption rate was almost levelled off owing to the drop in the metal ion concentration as well as the overloading of the active sites of the adsorbent.^28–30^

As can be deducted from Fig. 5(c), elevating the concentration of the target metal ion from 1 to 4 mg L^−1^ a remarkable decline in the efficiency of adsorption was noticed. The reverse relation between the analyte concentration and the adsorption efficiency is explained by the fact that there are a limited number of active sites available on the applied adsorbent following the saturation phenomenon.^31^

### 3.5. Isotherm studies

Three notable isotherm models, namely the Freundlich, Langmuir, and Temkin along with their corresponding equations were applied to interpret the isotherm behaviors of the adsorption process. As evident from Table 1, the *R*^2^ value for the Langmuir model (*R*^2^ > 0.99) is the highest compared to the other respective models. This finding indicates that the data obtained are in well consistent with the Langmuir model. Additionally, the results demonstrated that Hg(ii) ions were in shape of monolayer and orderly adsorbed on the applied adsorbent homogeneous surface.^32^ Meanwhile, the highest capacity of adsorption derived from the model of Langmuir was determined to be 17.68 mg g^−1^ for Hg(ii) ions. In a further related development, the separation factor (*R*_L_) for the Langmuir model was applied to estimate the favorability of the process of adsorption (*R*_L_ > 1 denoted as unfavorable, 0 < *R*_L_ < 1 denoted as favorable, *R*_L_ = 0 denoted as irreversible, and *R*_L_ = 1 denoted as linear).^33^ The calculated *R*_L_ values matched with 0 < *R*_L_ < 1 range, which indicates that the ions of interest were favorably adsorbed on adsorbent.

**Table 1:** Kinetic and isotherm values/parameters for the adsorption of Hg(ii)

Model	Equation	Nomenclature	Parameters	Hg(ii)
**Isotherms**				
Langmuir	*C*_e_/*q*_e_ = *C*_e_/*Q*_m_ + 1/*K*_a_*Q*_m_	The slope and intercept of the linear plot of *C*_e_/*q*_e_*versus C*_e_ give *Q*_m_ and *K*_a_, respectively	*Q*_m_ (mg g^−1^)	17.68
			*K*_L_ (L mg^−1^)	31.10
			*R*_L_	0.01–0.03
			*R* ^2^	0.997
Freundlich	ln *q*_e_ = (1/*n*)ln *C*_e_ + ln *K*_F_	The slope and intercept of the linear plot of ln *q*_e_*versus* ln *C*_e_ give 1/*n* and *K*_F_, respectively	*n*	4.60
			*K*_F_ (L mg^−1^)	1.06
			*R* ^2^	0.876
Temkin	*q*_e_ = *B*_l_ ln *C*_e_ + *B*_l_ ln *K*_T_	*B*_1_ and *K*_T_ are calculated from the slope and intercept of the linear plot of *q*_e_ against ln *C*_e_, respectively	*B*_l_	298.51
			*K*_T_ (L mg^−1^)	9.67
			*R* ^2^	0.88

**Kinetics**				
Pseudo-first-order	ln(*q*_e_ − *q*_*t*_) = −*k*_1_*t* + ln(*q*_e_)	The slope and intercept of the linear plot of ln(*q*_e_ − *q*_*t*_) *versus t* give *k*_1_ and *q*_e_, respectively	*k*_1_ (min^−1^)	0.066
			*q*_e_ (mg g^−1^)	4.90
			*R* ^2^	0.9969
Pseudo-second-order	*t*/*q*_*t*_ = *t*/*q*_e_ + 1/(*k*_2_*q*_e_)^2^	The slope and intercept of the linear plot of *t*/*q*_*t*_*versus t* give *q*_e_ and *k*_2_, respectively	*k*_2_ (g mg^−1^ min^−1^)	0.29
			*q*_e_ (mg g^−1^)	0.937
			*R* ^2^	0.9988

### 3.6. Kinetics of adsorption

Generally, kinetics has been marked as being one of the most important factors for the description of the efficiency of an adsorption process. It is thought that rapid interactions between the adsorbent and the adsorbed analyte dominate the kinetics of adsorption in aqueous solutions. To describe the adsorption of the analyte of concern onto the applied adsorbent, the models of pseudo-1st order and pseudo-2nd order were individually considered.

The parameters and the corresponding regression correlation coefficient values (*R*^2^) with regard to the applied kinetic models are tabulated in Table 1. The results clearly revealed that the pseudo-2nd-order kinetic model (*R*^2^ > 0.998) is well fitted to describe the adsorption efficiency of Hg(ii) ions. It is highly assumed that the strong coordination sites located inside the cavities of the MOF-based modified adsorbent results in the strong interactions between the adsorbent and Hg(ii) ions. It seems that during the adsorption process, the dominant phenomenon involves the chelation of Hg(ii) ions with the –C=S functional group of the adsorbent through the soft–soft interactions (Hg(ii) ions and –C=S group) derived from the HSAB concept.^34^

### 3.7. Recycling of adsorbent

**Fig. 6:**
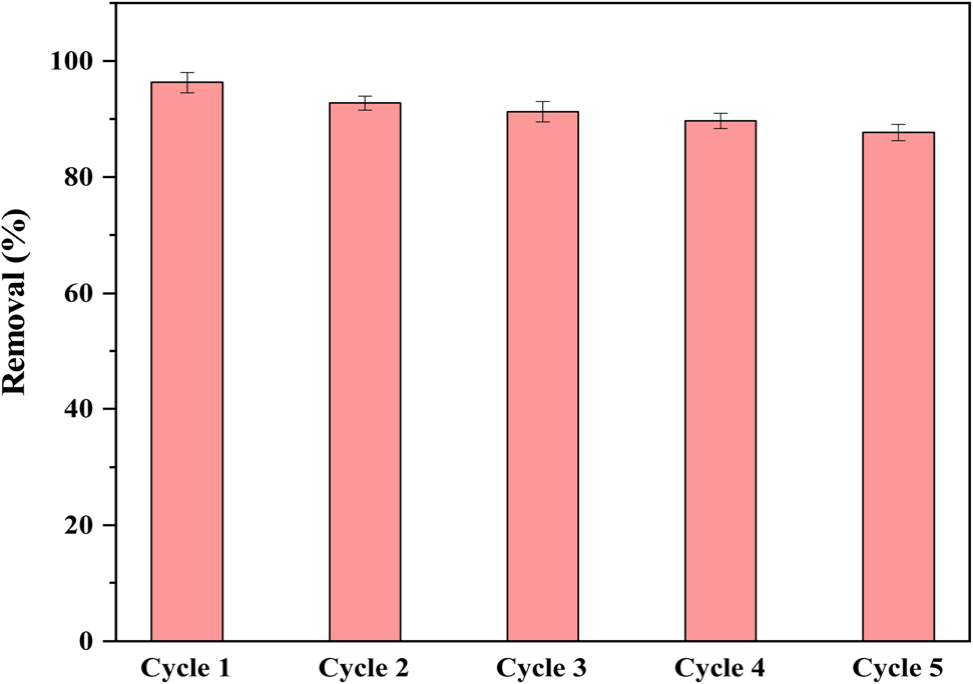
Reusability of the applied adsorbent over 5 cycles of use.

Regeneration/recycling is a critical parameter for choosing an effective and suitable adsorbent in water treatment systems. For this purpose, the adsorbent was subjected to the recycling test to determine the level of decline in the adsorbent performance after several reuse. The adsorption/desorption performance after five cycles of use was depicted in Fig. 6. As demonstrated, the adsorbent performance remained almost unchanged after five cycles of use, which indicates a high level of efficiency for the applied adsorbent. As observed within Fig. 6, the adsorption efficiencies for the target metal ion exhibited a tiny drop of 8.6% over the five cycles of use, which seems to be relatively satisfactory. The mentioned fall in the efficiency is highly likely attributed to the mass loss and/or the suppression of the applied adsorbent active sites.

### 3.8. A comparative-based study

In this section, a number of previously reported works on the adsorption of Hg(ii) ions in the literature were summarized in Table 2.^35–39^ As can be deduced, the efficiency and merits of this research-based study with regard to the adsorption and removal of Hg(ii) ions are fairly comparable with the other studies.

**Table 2:** Comparative study on the adsorption of Hg(ii) ions

Adsorbent	*C* (mg L^−1^)	Time	*R* (%)	References
Fe_3_O_4_@MOF	—	120 min	99	35
Magnetic/CoFe_2_O_4_@SiO_2_-SH	40	12 h	99	36
MXene	100	24 h	99.8	37
Biochar/NiFe_2_O_4_/Ag_3_PO_4_	10	90 min	98.5	38
UiO66-BAT-SH	100	180 min	97.2	39
MOF-based modified adsorbent	1	40 min	96.3	This work

## 4. Conclusion

In this study, initially the synthesis of a new MOF-based adsorbent featuring high porosity and oriented cavities was accomplished followed by the characterization by several instrumental analyses. Then, the effects of various influential parameters affecting the adsorption efficiency were investigated in details and the corresponding optimized values were established. The applied adsorbent exhibited fast and effective adsorption behavior toward the ions of Hg(ii) mainly through the chelation on the basis of the HSAB concept. As a result, the utmost removal efficiency of 96.3% for Hg(ii) was obtained. In addition, the adsorption process well fitted to the Langmuir isotherm and pseudo 2nd-order kinetics models. Finally, reuse of the applied adsorbent for five consecutive tests showed a tiny fall in the removal efficiency for target ion. It is thought that versatile MOF-based chemically modified adsorbents could be potentially applied in the water treatment processes for the successful removal of heavy metal ions from various water sources.

## Conflicts of interest

There are no conflicts to declare.

## Supplementary Material

## References

[cit1] Khan F. S. A., Mubarak N. M., Tan Y. H., Khalid M., Karri R. R., Walvekar R., Abdullah E. C., Nizamuddin S., Mazari S. A. (2021). J. Hazard. Mater..

[cit2] Lofrano G., Carotenuto M., Libralato G., Domingos R. F., Markus A., Dini L., Gautam R. K., Baldantoni D., Rossi M., Sharma S. K. (2016). Water Res..

[cit3] Velusamy S., Roy A., Sundaram S., Kumar Mallick T. (2021). Chem. Rec..

[cit4] Hong Y.-j., Liao W., Yan Z.-f., Bai Y.-c., Feng C.-l., Xu Z.-x., Xu D.-y. (2020). J. Chem..

[cit5] Briffa J., Sinagra E., Blundell R. (2020). Heliyon.

[cit6] Idris M., Kolo B., Garba S., Waziri I. (2013). Bull. Environ., Pharmacol. Life Sci..

[cit7] Nagar A., Pradeep T. (2020). ACS Nano.

[cit8] Yang X., Wan Y., Zheng Y., He F., Yu Z., Huang J., Wang H., Ok Y. S., Jiang Y., Gao B. (2019). Chem. Eng. J..

[cit9] Kökçam-Demir Ü., Goldman A., Esrafili L., Gharib M., Morsali A., Weingart O., Janiak C. (2020). Chem. Soc. Rev..

[cit10] Zhu Q.-L., Xu Q. (2014). Chem. Soc. Rev..

[cit11] Rouhani F., Rafizadeh-Masuleh F., Morsali A. (2019). J. Am. Chem. Soc..

[cit12] Shi Z., Xu C., Guan H., Li L., Fan L., Wang Y., Liu L., Meng Q., Zhang R. (2018). Colloids Surf., A.

[cit13] Lu F., Astruc D. (2020). Coord. Chem. Rev..

[cit14] Hasan Z., Jhung S. H. (2015). J. Hazard. Mater..

[cit15] Jeirani Z., Niu C. H., Soltan J. (2017). Rev. Chem. Eng..

[cit16] Liu J., Wang Y. (2023). Molecules.

[cit17] Wen M., Li G., Liu H., Chen J., An T., Yamashita H. (2019). Environ. Sci.: Nano.

[cit18] Chen L. and Hong M.-C., Design and Construction of Coordination Polymers, John Wiley & Sons, 2009

[cit19] Janiak C. (2003). Dalton Trans..

[cit20] Kitagawa S., Uemura K. (2005). Chem. Soc. Rev..

[cit21] Barea E., Montoro C., Navarro J. A. (2014). Chem. Soc. Rev..

[cit22] Esrafili L., Gharib M., Morsali A. (2019). Dalton Trans..

[cit23] Aragay G., Pons J., Merkoçi A. (2011). Chem.
Rev..

[cit24] Babel S., Kurniawan T. A. (2003). J. Hazard. Mater..

[cit25] Aghajanzadeh M., Zamani M., Molavi H., Khieri Manjili H., Danafar H., Shojaei A. (2018). J. Inorg. Organomet. Polym. Mater..

[cit26] Fotovat H., Khajeh M., Oveisi A. R., Ghaffari-Moghaddam M., Daliran S. (2018). Mikrochim. Acta.

[cit27] Dehghani M. H., Salari M., Karri R. R., Hamidi F., Bahadori R. (2021). Sci. Rep..

[cit28] Liu H., Liu W., Zhang J., Zhang C., Ren L., Li Y. (2011). J. Hazard. Mater..

[cit29] Jafari K., Heidari M., Rahmanian O. (2018). Ultrason. Sonochem..

[cit30] Ozer C., Imamoglu M., Turhan Y., Boysan F. (2012). Toxicol. Environ. Chem..

[cit31] Dovi E., Aryee A. A., Kani A. N., Mpatani F. M., Li J., Li Z., Qu L., Han R. (2021). J. Environ. Chem. Eng..

[cit32] Ali I., Afshinb S., Poureshgh Y., Azari A., Rashtbari Y., Feizizadeh A., Hamzezadeh A., Fazlzadeh M. (2020). Environ. Sci. Pollut. Res..

[cit33] Niri M. V., Mahvi A. H., Alimohammadi M., Shirmardi M., Golastanifar H., Mohammadi M. J., Naeimabadi A., Khishdost M. (2015). J. Water Health.

[cit34] LoPachin R. M., Gavin T., DeCaprio A., Barber D. S. (2012). Chem. Res. Toxicol..

[cit35] Ke F., Jiang J., Li Y., Liang J., Wan X., Ko S. (2017). Appl. Surf. Sci..

[cit36] Zhu H., Shen Y., Wang Q., Chen K., Wang X., Zhang G., Yang J., Guo Y., Bai R. (2017). RSC Adv..

[cit37] Hu X., Chen C., Zhang D., Xue Y. (2021). Chemosphere.

[cit38] Kaveh R., Mortazavi M., Alijani H., Abdouss M., Dehkalani A. S., Mazinani S. (2024). J. Solid State Chem..

[cit39] Huang X., Zhao M., Xu M., Hu J., Wang J., Miao X., Xie D. (2024). J. Mol. Struct..

